# A Prospective Study to Evaluate the Association of Sensory Dysregulation and Adverse Pathologic Features in Oral Tongue Cancers

**DOI:** 10.1007/s12663-024-02410-2

**Published:** 2024-12-10

**Authors:** Arjun Gurmeet Singh, Mihir Dani, Shwetabh Sinha, Rathan Shetty, Poonam Joshi, Sudhir Nair, Pankaj Chaturvedi

**Affiliations:** 1 Department of Head and Neck Oncology, Tata Memorial Center and HBNI, Mumbai, India; 2 Department of Radiation Oncology, Tata Memorial Center and HBNI, Mumbai, India

**Keywords:** Sensory dysregulation, Oral cancer, Tongue cancer, Recurrence, Survival

## Abstract

**Objectives:**

To correlate sensory dysfunction with adverse pathologic factors for tongue cancers and determine the survival prognosticators.

**Methods:**

We prospectively collected data on the pain sensation (Brief Pain Inventory), gustation (NIH Taste Intensity Test), proprioception (Two-point and light touch discrimination) from patients with squamous carcinoma of oral tongue, surgically treated between July 2021 and September 2022, and stratified them based on pT stage. The pathologist was blinded to the clinical data and correlation with the sensory dysregulations was obtained using a multivariate analysis. Factors predicting overall survival (OS) and disease specific survival (DSS) were calculated using Kaplan–Meier method.

**Results:**

100 patients were followed up with a median of 13 months. Radiating pain predicted advanced disease, extranodal extension (ENE) and perineural invasion (PNI). Loss of sweet and salty taste were associated with advanced disease, Depapillation and PNI. Among early tumors, 26% had taste alterations, with loss of sweet taste significantly associated with Depapillation and PNI. Loss of light touch was significantly associated with presence of PNI, while loss of two-point discrimination was associated with larger tumors, PNI and poor grade. The OS was significantly reduced when pain scores were moderate to severe at their least within 24 h from interview and loss of light touch.

**Conclusion:**

Radiating pain, sweet and salty taste dysregulation and loss of sensory discrimination have a strong correlation with PNI and impact survival.

## Introduction

Oral cancer is one of the most prevalent cancer sites emerging as a global public health concern. Its incidence, prevalence and distribution vary across regions of the world, although a large proportion is contributed by the South Asian regions [1]. This incidence is projected to see a 16% increase over the next 5 years, with almost half of the global deaths being contributed form the Indian subcontinent alone [1]. At the same time, it is one of the most preventable forms of cancer and one that can be easily detected at an early-stage owing to its accessibility. Despite this, a large proportion of patients present at an advanced stage due to a variety of reasons which negatively impacts survival outcomes [2]. Despite our current understanding of what we refer to as “early” oral cancers, a proportion tend to recur in the long term after completing the current form of conventional treatment [3]. Out of these, cancers of the tongue have been hypothesized to be more aggressive compared to the buccal cavity [4]. In an effort to improve outcomes for both survival and quality of life, various prognostic factors have been studied to modify management options. The presence of nodal metastasis, perineural invasion and margin status have been found to be more consistently reported [5]. Ironically, these are pathologic factors that are difficult to identify on preoperative biopsy, precluding their implementation in routine practice. Hence, clinical signs and symptoms specific to the site of cancer are useful contributors in determining the course of management for patients.

Outside of survival, pain and dysfunction are the primary concerns for oral tongue cancer patients [6]. These concerns can be significantly heightened in some as the tongue is a digestive organ which functions by facilitating mastication and assisting speech and swallowing. Finer functions such as sensation and taste are driven by even more complex processes [7]. Moreover, these cancers are known to spread in a submucosal manner making mapping of the disease difficult [8]. Due to this, it can also involve deeper structures which might not be very clinically apparent. Hence, it would be judicious to assume that even a slight abnormality in these complex processes could indicate an underlying pathologic process that could help us prognosticate tongue cancers better. Hence, the aim of our study was to correlate the dysfunction in sensory modalities specific to the oral tongue (gustatory, proprioception and pain referral) with the presence of adverse pathologic factors and determine which of the clinical factors predicts poor survival outcomes.

## Material and Methods

### Study Design

The academic investigators belonging to the Head and Neck Disease Management Group at Tata Memorial Center, Mumbai, designed and performed the study. The primary objective was to determine the correlation between the dysfunction in gustatory, proprioception and pain with the presence of adverse pathologic factors in tongue cancers, and their impact on survival outcomes. This single-center prospective clinical study was initiated after obtaining approval form the institutional ethics committee at Tata Memorial Center, Mumbai for recruiting 100 patients.

### Enrolment Criteria

The eligibility criteria included patients between 18 and 75 years with histologically proven treatment naïve invasive squamous cell carcinoma of the tongue that were planned to undergo definitive surgical management at our institute from July 2021 to September 2022. We excluded patients who had any previous treatment in the head and neck region, and any comorbidity or associated potentially malignant disorder that could possibly influence the presence of papillary atrophy on tongue. Patients having any history of mental illness (depression, anxiety, etc.) and taking medication for the same, those taking any form of week or strong opioid analgesics as per the WHO pain scale ladder for a period greater than 1 week prior to assessment, those having a history of other diseases and conditions, where taste sensation is disturbed (COVID-19, Sjogren’s syndrome, radiotherapy, etc.) and presence of trismus and/or ankyloglossia that hindered local assessment of the tongue were excluded from the study. The subjects were stratified into two groups, i.e., early (pT1–T2) and late (pT3–T4), based on the pathologic size of tumors.

### Study Procedures

Once the consent was obtained, the investigator/coordinator obtained relevant data at bedside or in the outpatient department. The following symptoms were measured using specific tools:

#### Pain

The validated English and Hindi versions of the Brief Pain Inventory (BPI) incorporating a conventional tongue diagram was used to quantify the severity of pain [9]. The BPI also assessed the impact of pain on daily function, location of pain, pain medications and amount of pain relief in the past 24 h or the past week from date of assessment. We used the short version of the BPI consisting of 9 questions that covered occurrence of pain, areas of pain, rating the pain at its worst and its least in the last 24 h of assessment, specifying the average pain level, current level of pain, treatments or medications being currently taken, percentage of pain relief form medications in the past 24 h and how much has the pain interfered in specific areas of life in the last 24 h from assessment.

#### Taste

The taste sensations were objectively measured based on the NIH toolbox protocol for Taste Intensity Test through a generalized labeled magnitude scale (gLMS) ranging from (no sensation, barely detectable, week, moderate, strong, very strong and strongest sensation of any kind) [10]. The patient was explained about the procedure and a laminated picture card was displayed before starting the procedure. Patients were not allowed to have any food or drinks at least 30 min before the test. At the start of the test, they were asked to rinse and spit their mouth twice with water at room temperature. The patient was then asked to remove their tongue and a tastant on a cotton swab was brushed along the nondiseased part of the lateral tongue running through the tip, and surrounding the disease. The patient was asked to score the taste sensation based on the scoring of the gLMS. Patient was asked to rinse and spit twice with water after each tastant was assessed. Tastants were prepared as per the NIH toolbox protocol and included sucrose solution (Sweet), sodium chloride solution (Salty), quinine hydrochloride solution (Bitter), citric acid solution (Sour) and monosodium glutamate solution (Umami) in distilled water [10].

#### Sensory Loss

Light touch was assessed with the cotton wisp test, where the examiner moved a filament near, but not touching, the tongue so that extrasensory information would remain constant [11]. Next, the thin wisp of cotton was applied to the tip, lateral border and dorsum, where the patient would provide a ‘yes’ (perceived touch) or ‘no’ (did not perceive touch) response [11]. Two-point discrimination was measured by presenting either one or two points to the tongue and asking the patient to distinguish between them [11]. The distances were calibrated by presenting one point and two points to the tip and the normal (non-cancer) side of the tongue, and indicating how many points were presented by show of fingers. A caliper was used to calculate the least distance between two discernible points.

#### Pathology

The pathologist was blinded to the clinical data captured on pain, taste and sensory loss. The final pathology variables (tumor size, nodal metastasis, grade of differentiation, margin status, depth of invasion (DOI), underlying muscle involvement, dominant pattern of invasion (POI), peritumoral lymphocytic infiltrate, extranodal extension (ENE), perineural invasion (PNI) and lymphovascular extension (LVE)) were reported as per the institute’s synoptic reporting system that follow the standard CAP, RCPATH and ICCR guidelines on reporting of various core data and noncore data elements.

### Statistical analysis

The data was analyzed using SPSS 28.0 (IBM, Armonk, NY). To identify the correlation of adverse pathologic factors and the presence of sensory alterations, a multivariate analysis was performed using a binary logistic regression model and a two-way ANOVA. Disease failure was documented after clinical, histopathological or radiological confirmation. The site of disease failure was noted as local, regional or distant in nature. Date of surgery was considered as baseline for calculation of the overall survival (OS) and disease specific survival (DSS). The patients were followed up till April 2023 and the Kaplan–Meier method and log-rank tests were used to calculate survival outcomes.

## Results

Out of the 673 tongue cancer patients screened, 103 patients were enrolled in the study. Three patients did not undergo surgery after enrolling in the study, the data of which we have excluded from analysis as the pathology data would not be available. All patients had a history of tobacco and/or areca nut use. The demographic details are shown in Table 1. The median follow-up time was 13 months ± 4.3 months. The factors that predicted poor OS were higher stage of tumors (*p*-0.008) and presence of PNI (*p*-0.004), while those for poor DSS were higher stage of tumors (*p*-0.042).

**Table 1:** Pathologic factors predicted by presence of radiating pain

	Radiating yes	Radiating no	*p* value
*Gender*			
Male	20 (25.6)	58 (74.4)	
Female	2 (9.1)	20 (90.9)	0.145
*Age*			
48 below	14 (28)	36 (72)	
48 above	8 (16)	42 (84)	0.227
*Depapillation*			
Present	18 (26.5)	50 (73.5)	
Absent	4 (12.5)	28 (87.5)	0.130
*pT*			
Early	6 (8.8)	62 (91.2)	
Advanced	16 (50)	16 (50)	< 0.001
*pN*			
Absent	5 (8.5)	54 (91.5)	
Present	17 (41.4)	24 (58.5)	< 0.001
*ENE*			
Present	11 (52.4)	10 (47.6)	
Absent	11 (13.9)	68 (86.1)	< 0.001
*LVI*			
Present	2 (66.7)	1 (33.3)	
Absent	20 (20.6)	77 (79.4)	0.121
*PNI*			
Present	13 (36.1)	23 (63.9)	
Absent	9 (14.1)	55 (85.9)	0.022
*DOI*			
Below 10 mm	5 (8.2)	56 (91.8)	
Above 10 mm	17 (43.6)	22 (56.4)	< 0.001
*Grade*			
WDSCC/MDSCC/PDSCC	13 (17.3)	62 (82.7)	
	9 (36)	16 (64)	0.091
*POI*			
3,4	14 (16.5)	71 (83.5)	
5,6	8 (53.3)	7 (46.7)	0.004

### Pain

Based on the BPI scoring sheet, the worst intensity of pain was experienced by 82% of the participants within 24 h from assessment, while the least painful intensity was seen in 67%. The mean pain score was 4.45 ± 1.76. At the time of assessment, 70% had mild intensity of pain, and 60% reported a mild relief of pain after taking medication. The interference of pain in daily work for severely affected general activity in 57%, mood in 49%, walking ability in 50%, normal work in 57%, relation with other people in 47%, sleep in 63% and enjoyment of life in 64% of participants. There were no statistically significant differences in the BPI scores among early and advanced stage tumors, except in the domain of relations with others (*p*-0.001). The presence of radiating pain was significantly more in late than early-stage tumors (19% vs. 3%, *p*-0.001). Presence of radiating pain predicted greater pT and pN, ENE, PNI, POI in univariate analysis and multivariate analysis. (Table 1). The OS was significantly reduced when pain scores were moderate to severe at their least within 24 h from interview (p-0.008). A worsening trend of OS was seen with the presence of severe pain at presentation (*p*-0.07). The DSS was not significantly impacted by any of the pain variables. However, a declining trend of DSS was seen if a 40% relief of pain was not seen with pain medications (*p*-0.06).

### Taste

**Fig. 1:**
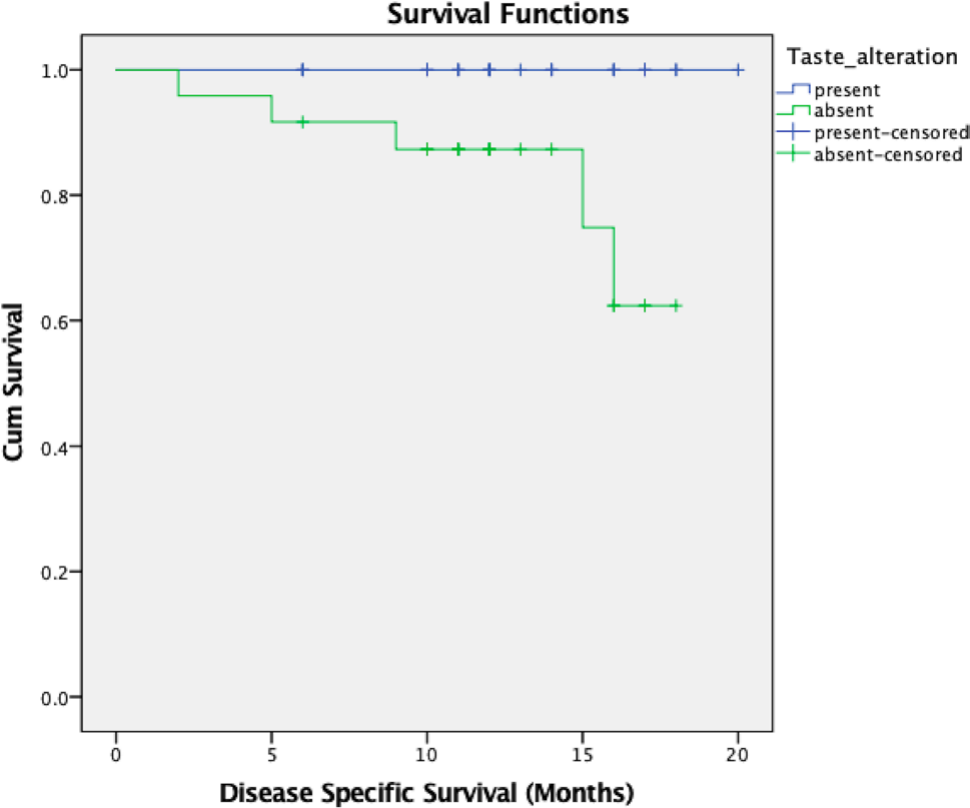
Disease specific survival in the presence of any alteration in taste among early cancers

Alterations in taste were present in 64% of the group, with a significant proportion being advanced stage tumors (38% vs. 26%, *p*-0.021). Loss of sweet taste was significantly associated with advanced pT and pN stage disease, depapillation and PNI. Similar results were seen for loss of salty taste, except for in pN stage. Loss of sour taste was associated with advanced pT stage, depapillation, ENE, PNI, and POI, while loss of umami taste was seen with PNI (Table 2). Considering only early-stage tumors, 26% had some form of taste alteration. Loss of sweet taste was in significant association with the presence of depapillation (*p*-0.014) and PNI (*p*-0.010). Loss of sour taste was associated with worse POI (*p*-0.048). Within PNI among early cancers, major PNI had a significant association with taste alterations than minor PNI (*p*-0.004). Alterations in other tastes were not associated with poor pathologic features in clinically early tongue cancers. However, the DSS was significantly impacted with the presence of any alteration in taste among early cancers (*p*-0.014) (Fig. 1).

**Table 2:** Association of taste alterations and pathologic factors

	Sweet			Salty			Sour			Bitter			Umami		
	Pres	Abs	sig	Pres	Abs	sig	Pres	Abs	sig	Pres	Abs	sig	Pres	Abs	sig
*Gender*															
Male	35	43		26	52		29	49		16	62		12	66	
Female	8	14	.627	6	16	.796	6	16	.456	3	19	.555	3	19	1.00
*Age*															
48 below	25	25		17	33		19	31		15	35		9	41	
48 above	18	32	.225	15	35	.830	16	34	.675	4	46	.009	6	44	.577
*Depapillation*															
Present	35	33		28	40		31	37		16	52		12	56	
Absent	8	24	.017	4	28	.005	4	28	.001	3	29	.108	3	29	.375
*pT*															
Early	24	44		14	54		15	53		14	54		8	60	
Advanced	19	13	.031	18	14	.001	20	12	.001	5	27	.785	7	25	.232
*pN*															
early	20	39		16	43		17	42		10	49		7	52	
advanced	23	18	.040	16	25	.276	18	23	.139	9	32	.608	8	33	.394
*ENE*															
Present	12	9		10	11		13	8		5	16		6	15	
Absent	31	48	.214	22	57	.114	22	57	.009	14	65	.539	9	70	.080
*LVI*															
Present	2	1		1	2		1	2		1	2		0	3	
Absent	41	56	.576	31	66	1.00	34	63	1.00	18	79	.472	15	82	1.00
*PNI*															
Present	24	12		20	16		20	16		5	31		10	26	
Absent	19	45	.001	12	52	.001	15	49	.002	14	50	.429	5	59	.017
*DOI*															
Below 10 mm	21	40		13	48		13	48		11	50		6	55	
Above 10 mm	22	17	.039	19	20	.008	22	17	.001	8	31	.798	9	30	.088
*Grade*															
Well/mod	29	46		22	53		22	53		12	63		8	67	
Poor	14	11	.163	10	15	.333	13	12	.053	7	18	.239	7	18	.051
*POI*															
3,4	34	51		25	60		26	59		14	71		11	74	
5,6	9	6	.168	7	8	.232	9	6	.040	5	10	.154	4	11	.233

### Sensory Loss

**Fig. 2:**
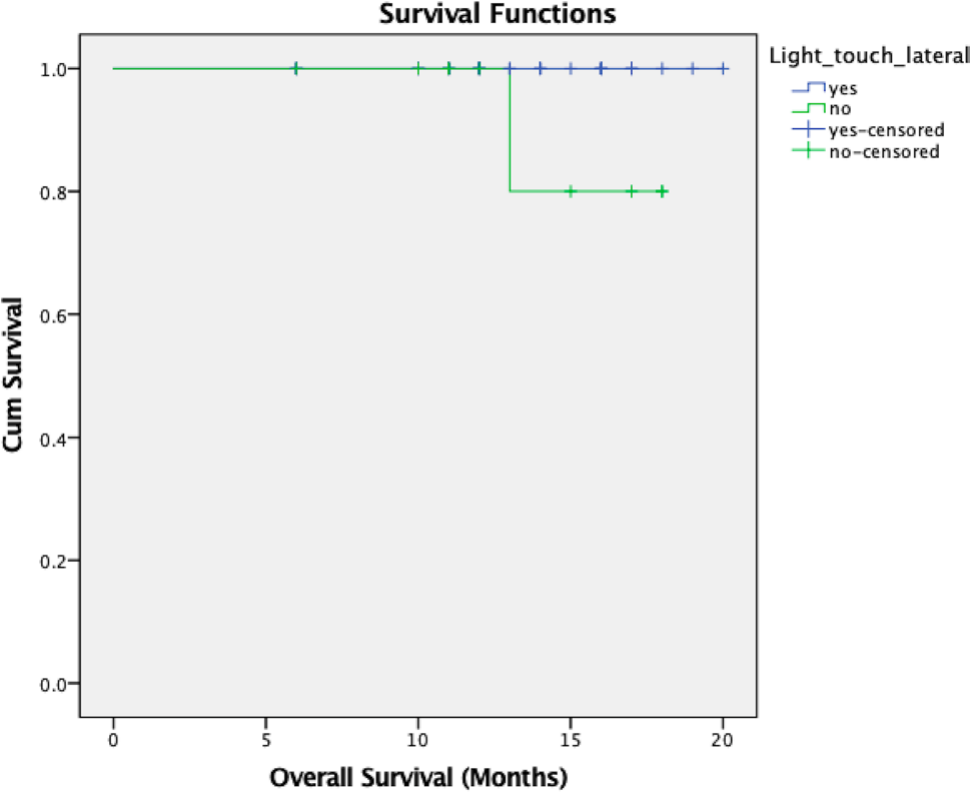
Overall survival for the loss of light touch on lateral border among entire cohort

Light touch sensation was lost at the tip in 1%, laterally in 16% and on the dorsum in 11% of patients. Loss of light touch on the lateral aspect was seen to be significantly associated with PNI. Two-point discrimination at the tip was present in 91% of the cases with a median discrimination distance of 4 mm ± 1.1. On the non-cancer lateral tongue edge, the two-point discrimination was present in 99% of patients with a median of 5 mm ± 0.8. Loss of two-point discrimination at the tip was associated with tumors crossing midline, advanced pT, presence of PNI, higher DOI and poorer grade of tumor. (Table 3) Among the early cancers, loss of light touch at the lateral border of tongue was significantly associated with presence of PNI (*p*- < 0.001), while loss of two-point discrimination at the tip was associated with pT2 tumors (*p*- < 0.001), PNI (*p*-0.010) and poor grade of differentiation (*p*- < 0.001). Moreover, loss of light touch on lateral border was significantly associated with poor OS (*p*-0.05) (Fig. 2).

**Table 3:** Association of sensory loss and poor pathologic factors

	Light touch tip			Light touch lateral			Light touch dorsum			Two-point tip			Two points non cancer		
	Pres	Abs	sig	Pres	Abs	sig	Pres	Abs	sig	Pres	Abs	sig	Pres	Abs	sig
*Gender*															
Male	77	1		64	14		69	9		9	69		1	77	
Female	22	0	1.00	20	2	.512	20	2	1.00	0	22	.200	0	22	1.00
*Age*															
48 below	49	1		38	12		44	6		7	43		0	50	
48 above	50	0	1.00	46	4	.054	45	5	1.00	2	48	.160	1	49	1.00
*Depapillation*															
Present	67	1		54	14		59	9		8	60		0	68	
Absent	32	0	1.00	30	2	.084	30	2	.495	1	31	.265	1	31	.320
*Crossing midline*															
Yes	10	0		9	1		8	2		3	7		0	10	
No	89	1	1.00	75	15	1.00	81	9	.302	6	84	.044	1	89	1.00
*pT*															
Early	68	0		58	10		59	9		1	67		1	67	
Advanced	31	1	.320	26	6	.771	30	2	.495	8	24	.001	0	32	1.00
*pN*															
Early	59	0		48	11		50	9		4	55		1	58	
Advanced	40	1	.410	36	5	.423	39	2	.192	5	36	.481	0	41	1.00
*ENE*															
Present	20	1		18	3		20	1		3	18		0	21	
Absent	79	0	.210	66	13	1.00	69	10	.450	6	73	.392	1	78	1.00
*LVI*															
Present	3	0		2	1		3	0		0	3		0	3	
Absent	96	1	1.00	82	15	.411	86	11	1.00	9	88	1.00	1	96	1.00
*PNI*															
Present	35	1		23	13		30	6		7	29		0	36	
Absent	64	0	.360	61	3	.001	59	5	.196	2	62	.010	1	63	1.00
*DOI*															
Below 10 mm	61	0		53	8		54	7		0	61		1	60	
Above 10 mm	38	1	.390	31	8	.404	35	4	1.00	9	30	.001	0	39	1.00
*Grade*															
WDSCC/MDSCC/	74	1		65	10		68	7		2	73		1	74	
PDSCC	25	0	1.00	19	6	.220	21	4	.460	7	18	.001	0	25	1.00
*POI*															
3,4	84	1		74	11		76	9		6	79		0	85	
5,6	15	0	1.00	10	5	.061	13	2	.669	3	12	.132	1	14	.150

## Discussion

The oral tongue is a complex organ with a specialized anatomy for taste and sensation [7]. Our hypothesis was to identify certain dysfunctions of taste and sensation that might appear early and correlate with adverse pathology features. To our knowledge, this is the first study in the literature studying these sensory alterations in tongue cancers and correlating them with survival outcomes. This information can help identify vital signs and symptoms early so that the management of tongue cancers can be improved.

A large majority of patients present to a medical or dental clinic with the initial symptoms of pain that leads to a diagnosis of oral cancer [12]. Several quality of life studies that have studied oral cancer-associated symptoms report a unifying conclusion that, other than survival, pain is the most important concern for these patients. In fact, a meta-analysis of 52 studies has shown that the highest prevalence of pain is seen in head and neck cancer, surpassing gynecological, gastrointestinal, lung and breast cancer [6]. This pain can be mild but has often been described as uncontrollable and creating a poor quality of life as they limit normal functions such as speech, swallowing, deglutition and interpersonal relations [13]. Hence, it is very important to understand and infer the presence or absence of pain and its quality. A large part of our cohort experienced their worst intensity of pain in the last 24 h from assessment, with a mild relief being achieved in more than half after non-opioid medication. Moreover, more than half of these had a disturbed general activity, normal work, sleep and enjoyment of life due to the level of pain. Traditionally, pain is known to increase with stage of disease, but we found no major differences in pain scores across tumor stage. Radiating pain on the other hand was associated with advanced stage of disease and PNI. Tongue cancers are often associated with pain which can be radiating to the pharynx and ear [14]. Moreover, we found that nonresponsive pain to analgesics had worse survival outcomes. Neural invasion has also been associated with increased rate of nodal metastasis, both of which alter the prognosis even in a conventionally staged early tumor [15]. Unique to oral cancer tumors, the dense trigeminal innervation effectively localizes pain at the primary site in contrast to tumors of other primary sites such as the gastrointestinal tract or pelvis, which tend to be more visceral [14]. In fact, surgical resection of these tumors has also been found to provide near-total pain relief, pointing to the association of microenvironment as the source of pain [16]. These results could signify a deep-rooted pain intrinsically associated with peripheral nervous system that could require greater analgesic support early on, irrespective of stage of disease. Presence of severe pain, and not just radiating pain, also points to a high correlation with perineural involvement.

Taste is a complex sense produced by the taste buds on the tongue [7]. Loss or alterations of taste have been linked to a number of diseases, but its association with tongue cancers has never been studied before [7]. More than half of the current cohort had alterations in taste sensation. Interestingly, none of these changes in taste were noticed by the patients until the study methodology was undertaken. This could suggest that these taste buds are sensitive to disease processes and could be used to detect changes early on, possibly even before clinical presentation. We also found that PNI invasion was significantly associated with loss of sweet, salty, sour and umami tastes. PNI has been associated with a poor survival prognosis and studies have also suggested to intensify treatment in its presence [15]. Hence, in the presence of these taste dysfunctions, it might be prudent to investigate the presence of any subclinical perineural spread and be aggressive in the surgical resection of the primary tumor considering a compartment resection.

When considering early-stage cancers, 26% had some form of taste alteration with sweet taste being significantly associated with presence of depapillation and PNI. Loss of papilation over the tongue has been shown to predict PNI in early cancers with a subsequently impact on survival [17]. Even with a limited follow-up period, the DSS was significantly impacted with the presence of alteration in taste among early-stage cancers. We also found that light touch sensation was lost at various parts of the tongue in a small proportion of patients. At the same time, the ability for discrimination between two points was maintained in a large majority. However, when absent in early cancers, it was significantly associated with PNI with a significant impact on overall survival. Alterations in taste sensations, especially sweet and salty, and loss of two-point discrimination might be an early indication of an aggressive disease owing to their strong association with PNI. Further research is needed to understand the tumor microenvironment and local biochemical processes that result in these clinical signs and adverse pathology features.

## Conclusion

Our study is the first to report novel prognostic information demonstrating the correlation between sensory dysregulation in taste and touch, and adverse pathology features such as perineural invasion. This information could aid in early diagnoses and management for oral tongue caners and improve survival outcomes.
